# Single-cell transcriptomics of staged oocytes and somatic cells reveal novel regulators of follicle activation

**DOI:** 10.1530/REP-22-0053

**Published:** 2022-06-17

**Authors:** Yu-Ying Chen, Daniela D Russo, Riley S Drake, Francesca E Duncan, Alex K Shalek, Brittany A Goods, Teresa K Woodruff

**Affiliations:** 1 Department of Obstetrics and Gynecology, Feinberg School of Medicine, Northwestern University, Chicago, Illinois, USA; 2 Ragon Institute of MGH, MIT, and Harvard, Cambridge, Massachusetts, USA; 3 Broad Institute of MIT and Harvard, Cambridge, Massachusetts, USA; 4 Institute of Medical Science and Engineering, Department of Chemistry, and Koch Institute for Integrative Cancer Research, MIT, Cambridge, Massachusetts, USA; 5 The Thayer School of Engineering, Dartmouth College, Hanover, New Hampshire, USA; 6 Department of Obstetrics and Gynecology, Michigan State University, East Lansing, Michigan, USA

## Abstract

**In brief:**

Proper development of ovarian follicles, comprised of an oocyte and surrounding somatic cells, is essential to support female fertility and endocrine health. Here, we describe a method to isolate single oocytes and somatic cells from the earliest stage follicles, called primordial follicles, and we characterize signals that drive their activation.

**Abstract:**

Primordial follicles are the first class of follicles formed in the mammalian ovary and are comprised of an oocyte surrounded by a layer of squamous pre-granulosa cells. This developmental class remains in a non-growing state until individual follicles activate to initiate folliculogenesis. What regulates the timing of follicle activation and the upstream signals that govern these processes are major unanswered questions in ovarian biology. This is partly due to the paucity of data on staged follicle cells since isolating and manipulating individual oocytes and somatic cells from early follicle stages are challenging. To date, most studies on isolated primordial follicles have been conducted on cells collected from animal-age- or oocyte size-specific samples, which encompass multiple follicular stages. Here, we report a method for collecting primordial follicles and their associated oocytes and somatic cells from neonatal murine ovaries using liberase, DNase I, and Accutase. This methodology allows for the identification and collection of follicles immediately post-activation enabling unprecedented interrogation of the primordial-to-primary follicle transition. Molecular profiling by single-cell RNA sequencing revealed that processes including organelle disassembly and cadherin binding were enriched in oocytes and somatic cells as they transitioned from primordial to the primary follicle stage. Furthermore, targets including WNT4, TGFB1, FOXO3, and a network of transcription factors were identified in the transitioning oocytes and somatic cells as potential upstream regulators that collectively may drive follicle activation. Taken together, we have developed a more precise characterization and selection method for studying staged-follicle cells, revealing several novel regulators of early folliculogenesis.

## Introduction

The ovarian follicle is the functional unit of the ovary and consists of the oocyte surrounded by supporting somatic cells. Proper folliculogenesis, or the process of follicle development, is required to support future fertility, endocrine function, and systemic health in female mammals. Prior to follicle formation, primordial germ cells proliferate via incomplete cytokinesis during mitotic division that results in clusters of oogonia connected by cytoplasmic bridges, collectively surrounded by somatic pre-granulosa cells (also termed the ovarian cysts) (Anderson *et al.* 2000, Molyneaux *et al.* 2001, Pepling & Spradling 2001). The oogonia then enter meiosis and differentiate into oocytes, which then arrest at the diplotene stage of prophase in meiosis I (Bullejos & Koopman 2004, Cordeiro *et al.* 2015*a*) before becoming individually encapsulated primordial follicles surrounded by pre-granulosa cells (Pepling & Spradling 2001). As the first class of follicle formed in an ovary, the fate of a primordial follicle is to either activate and initiate follicle growth, to die by attrition, or remain in a non-growing state. Before they activate and initiate the irreversible process of active follicle growth in preparation for ovulation, primordial follicles can remain in the non-growing state for days to months in mice or decades in humans (Tingen *et al.* 2009). Currently, the regulation of the activation process is not completely understood, and we have limited ability to identify those follicles that will be activated at precise times during the reproductive lifespan (Cordeiro *et al.* 2015*b*).

The number of primordial follicles in the ovary, termed the ovarian reserve, dictates the reproductive lifespan of females. This number at any given time is determined by the rate of follicle activation and depletion. The activation process involves the transition of a primordial follicle, in which a single oocyte is surrounded by a single layer of squamous pre-granulosa cells, to a primary follicle, in which the oocyte is surrounded by a single layer of differentiated cuboidal granulosa cells. This process is recognized phenotypically by two adjacent pre-granulosa cells asymmetrically growing in an apical direction at their shared membrane interface, collaboratively creating a ‘wedge-shape’ to the adjacent cells, and forming a transitioning follicle, as the activation signals activate in the oocyte (Mora *et al.* 2012, Hardy *et al.* 2018). Several signaling pathways including mTOR-PI3K-AKT-FOXO3 (John *et al.* 2008, Zhang *et al.* 2014) and the Hippo pathway (Kawamura *et al.* 2013, Kawashima & Kawamura 2018), oocyte-specific genes such as Nobox and Sohlh1 (Suzumori *et al.* 2002, Rajkovic *et al.* 2004, Pangas *et al.* 2006), and endocrine or nutrient factors such as anti-Müllerian hormone and oxygen levels (Zhang & Liu 2015, Shah *et al.* 2018, Zhang *et al.* 2021) are critical players in the activation process. However, there is a gap in our understanding of how these separate genes and pathways connect. We do not understand the ultimate upstream factors that trigger the above pathways or the mechanism underlying the selective and timed pattern of follicular activation across a female’s reproductive lifespan. Therefore, there is a need to study the process in the activating oocyte and somatic cells through an unbiased pathway and target-based approach.

Studies on primordial follicles traditionally involve their manipulation at the tissue or organ level or via single cells collected using animal-age- or oocyte size-specific selection criteria (Pan *et al.* 2005, Veselovska *et al.* 2015, Ford *et al.* 2021, Lv *et al.* 2021). These approaches preclude the ability to study cells in precise follicle stage-specific populations because both animal-age and size-specific cells are heterogeneous with respect to follicle stages. Downstream analyses including live staining, transcriptomic, or proteomic analysis on individual primordial follicle oocytes and somatic cells are challenging without an established protocol to isolate follicle-staged cells. In the current study, we describe a protocol for isolating primordial follicles and their associated single oocytes and pre-granulosa cells from neonatal mouse ovaries. The protocol involves three rounds of enzymatic treatments with liberase and DNase I, followed by physical agitation within each round to first collect intact primordial follicles. Single primordial follicle oocytes and pre-granulosa cells with high viability can then be collected with additional enzyme incubation. Isolated single cells profiled by RNA-sequencing yielded high-quality data and expressed key genes of interest. In addition, we characterized single cells within transitioning follicles collected by our isolation method which revealed early follicle activation pathways and potential upstream regulators that may drive the global shift during primordial follicle activation. Overall, our isolation method enables a precise characterization of murine primordial follicles and their associated oocytes and somatic cells.

## Materials and methods

Chemicals, reagents, and buffer were purchased from MilliporeSigma unless otherwise specified. All solutions were prepared using volume concentration (% v/v). Experiments were performed at room temperature unless otherwise noted.

### Animals

Female and male CD-1 breeders were purchased from Envigo (Indianapolis, IN, USA) and bred in house at the animal facility under Northwestern University Center for Comparative Medicine. Food (Teklad 2020X, Envigo) and water were given *ad libitum* and the mice were kept in a 14-h light: 10-h darkness cycle with constant temperature and humidity. Animals were treated in accordance with the National Institutes of Health Guide for the Care and Use of Laboratory Animals. All protocols were approved by the Northwestern University Institutional Animal Care and Use Committee (IACUC).

### Follicle counting and measurement

Ovaries were dissected out of the bursa of postnatal day 0, 2, 6, 12, 17, and 24 (P0, P2, P6, P12, P17, and P24) animals and fixed in Modified Davidson’s fixative (Electron Microscopy Sciences, Hatfield, PA, USA) for 16 h at 4°C for histology processing. After fixation, ovaries were washed in 50, 60, and 70% ethanol (Decon Labs Inc., King of Prussia, PA, USA) for 5 min each at 25°C. Ovaries were then dehydrated using a tissue processor (Leica Microsystems) before being embedded in paraffin blocks (Mercedes Scientific, Lakewood Ranch, FL, USA). Serial sections of the whole ovaries were cut at 5 μm (Leica Microsystems) and let dry at 25°C. Hematoxylin and eosin (H&E) staining was performed using an autostainer (Leica Microsystems) with standard protocol. Specifically, P0 and P2 ovarian sections were stained for 1 min, while P6-P24 ovarian sections for 9 min in hematoxylin (EKI, Joliet, IL, USA), before rinsing in acid ethanol (80% ethanol with 0.1% hydrochloric acid. Ethanol was prepared from 100% reagent alcohol from Mercedes Scientific. Hydrochloric acid was purchased from Thermo Fisher Scientific) for 6 seconds, blued in saturated lithium carbonate for 1 minute, counterstained in eosin (Astral Diagnostic, Logan Township, NJ, USA) for 1 minute, following dehydration and clearing. Ovarian sections were imaged on every fifth section with an EVOS cell imaging system (Thermo Fisher Scientific). For follicle quantification, only follicles showing clear nuclear (primordial follicle stage and prior) or nucleoli (growing follicles) staining were counted (Kim *et al.* 2015). Diplotene oocytes were distinguished by the presence of clear areas separating condensed chromatin in the oocyte nucleus (Dutta *et al.* 2016); follicles fully surrounded by one layer of squamous pre-granulosa cells were classified as primordial follicles; primary follicles were identified by a full layer of cuboidal granulosa cells, while secondary follicles with exact two layers of somatic cells. Follicles with three or more layers of somatic cells were classified as the multilayer stage. Once an antrum was observed, the follicles were counted as the antral stage. Follicles were classified as a stage before, if they did not meet the criteria fully (Supplementary Fig. 1, see section on supplementary materials given at the end of this article) (Bristol-Gould *et al.* 2006, Kim *et al.* 2015). The sum of each follicle classes was averaged for the counted sections, then multiplied by the total section number to obtain the total follicle number per ovary. To account for duplicating the count of small follicles, the total number of cystic oocytes, primordial follicles, and primary follicles were further divided by 2 (Bristol-Gould *et al.* 2006). In total, five to six ovaries were counted per age group. The size of the oocytes was measured by averaging two diagonal lengths on the same ovarian sections in ImageJ. Twenty-five to thirty oocytes were measured in each group.

### Follicle isolation

Eight to twelve P6 ovaries were dissected out of the bursa in Leibovitz's L-15 medium (Thermo Fisher Scientific) supplemented with 1 mg/mL fetal bovine serum (FBS, Thermo Fisher Scientific) and 50 U/mL penicillin–streptomycin (Thermo Fisher Scientific) for follicle isolation. Ovaries were transferred with a trimmed P1000 pipette tip into either a nine-well glass plate or a four-well dish (Thermo Fisher Scientific) with 500 μL Leibovitz's L-15 medium supplemented with 1 mg/mL poly(vinyl alcohol) (PVA, hereafter referred to as L-15/PVA), 30.8 μg/mL Liberase TM (Roche), and 456 U/mL DNase I (Worthington, NJ, USA) and incubated on a 37°C heated stage for 13 min with lid. The ovaries were then rinsed in 500 μL L-15/PVA before being transferred to 500 μL L-15/PVA supplemented with 50 μL/mL FBS and mechanically disrupted through repeated pipetting with a P200 pipettte set at 170 μL and a trimmed P200 tip (about 0.6 mm opening) for 4 min at 25°C. The process was followed by a second round of digestion in L-15/Liberase for 7 min, rinsing, and 4 min of mechanical pipetting (around 0.5 mm trimmed P200 tips), and the third round of digestion in L-15/ Liberase for 4 min, rinsing, and 4 min of mechanical pipetting (using a 0.4 mm trimmed P200 tip for 2 min following an untrimmed P200 tip for 2 min) where ovaries were being transferred across wells (Kniazeva *et al.* 2015). All transferring of ovaries was carried out using a P1000 trimmed pipette tip to avoid the transferring of the excess medium. After the third round of agitation, all three wells of L-15/PVA/FBS with the dispersed follicles were collected and passed through a 70-μm cell strainer (Corning) into a center well dish (Thermo Fisher Scientific) (Hornick *et al.* 2012, Laronda *et al.* 2014) (Supplementary Fig. 2). Quantification of total follicle number was carried out from the final follicle solution that was diluted 20×. To collect follicles, primordial, transitioning (follicles with partial squamous and partial cuboidal somatic cells), and primary follicles were selected by a mouth pipette with 50–75 μm stripper tips (Origio Inc., Charlottesville, VA, USA). Secondary follicles were selected by 100–125 μm stripper tips under a dissection scope with 11.25× objective or above (Nikon SMZ1500). Follicles were washed in three drops of 50 μL L-15/ PVA with 100 μL/mL FBS and staged under 20–40× objective (Leica DM IRB).

### Single oocyte and somatic cell isolation

**Figure 1:**
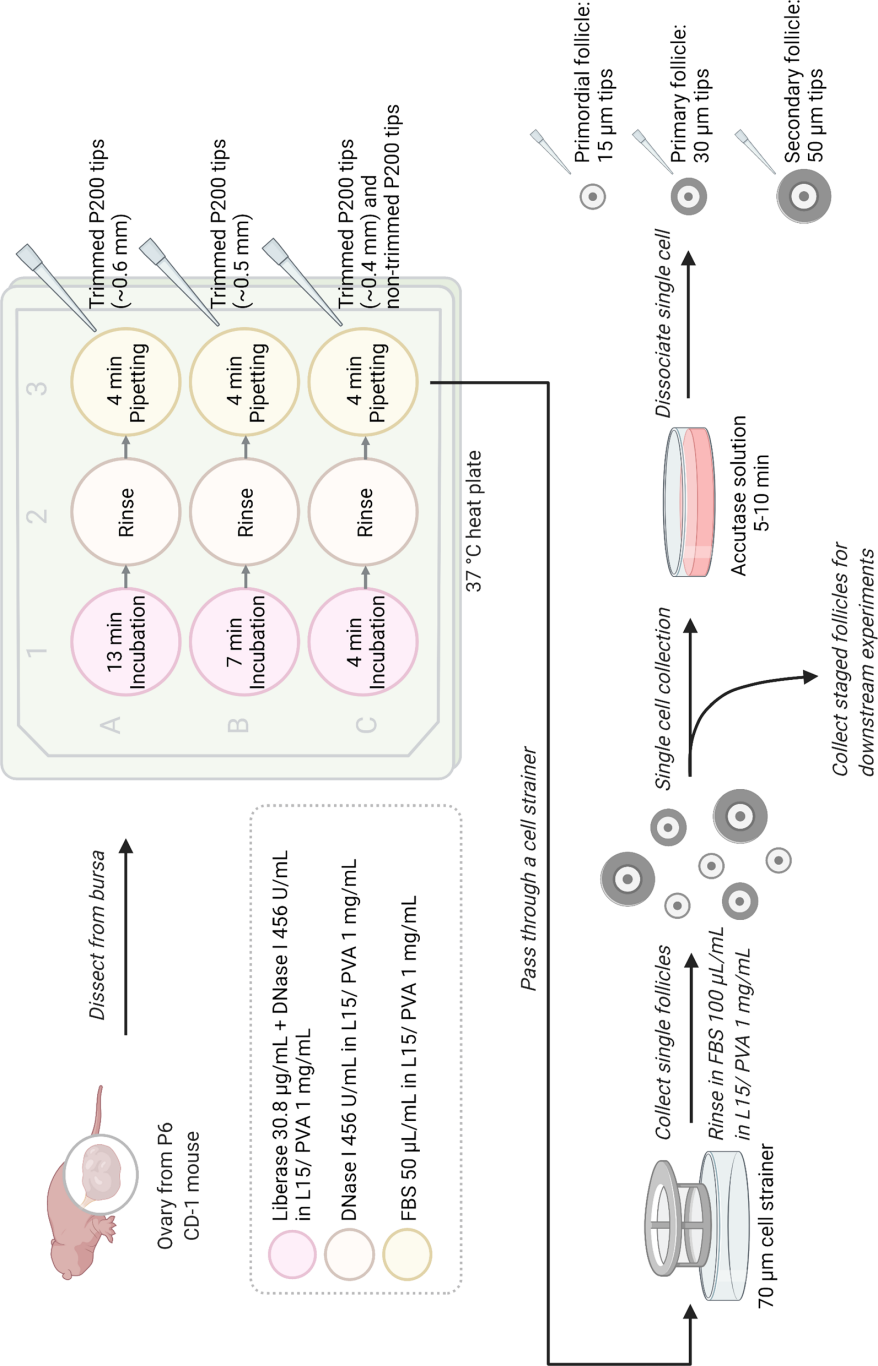
Illustration of the follicle and staged single-cell isolation protocol. The method involves three rounds of enzymatic digestions in liberase and DNase I, with a mechanical digestion following each round of enzymatic incubation. The isolated follicles can be subjected to further enzymatic digestion to dissociate the oocyte from individual pre-granulosa cells. Illustration created with BioRender.com.

For single oocyte and somatic cell collection, after determining the stages, follicles were incubated in Accutase solution (Stemcell Technologies, Canada) (Zhang *et al.* 2018) until somatic cells partially detached from each other. Specifically, primordial and transitioning follicles were incubated in Accutase solution for 5 min, and primary and secondary follicles were incubated in Accutase solution for 10 min on 37°C heated stage following agitation to dissociate somatic cells from the oocyte. Primordial and transitioning follicles were pipetted with 20 μm Pasteur pipette prepared by a micropipette puller (Sutter Instrument, Novato, CA, USA), primary follicle with a 30-μm Pasteur pipette and secondary follicle with a 50-μm stripper tip (Origio Inc.). Single cells were washed in three drops of 50 μL L-15/PVA with 100 μL/mL FBS. See Fig. 1 for follicle and single-cell isolation diagram.

### Follicle encapsulation and staining

Isolated follicles were fixed in 4% paraformaldehyde in Dulbecco’s phosphate-buffered saline (DPBS) at 4°C for 16 h. Afterward, follicles were rinsed in DPBS before being transferred to 50 μL 0.5% (w/v) alginate/DPBS solution on a piece of parafilm sheet (Xu *et al.* 2006, West *et al.* 2007). The alginate droplet was then slowly pushed into calcium solution (50 mM CaCl_2_ and 140 mM NaCl) for cross-linking. Once the alginate beads were set (about 5 min), the beads were stained briefly in 0.5% (w/v) alcian blue prepared in 0.25% acetic acid, embedded in paraffin block, sectioned and H&E stained as described above.

### Cell viability assay

Isolated oocytes and somatic cells were first rinsed in DPBS supplemented with 1 mg/mL PVA. Cell survival rate was then quantified by using the commercial Live and Dead Dye (Abcam) at a final concentration of 5× in DPBS/1 mg/mL PVA at 25°C for 10 min in the dark. Cells were then imaged by the EVOS cell imaging system, with live cells detected using the GFP channel and dead cells detected by the RFP channel (Supplementary Fig. 3). About 20 oocytes and 300 somatic cells were quantified for each follicle stages. The quantification was repeated four to five times.

### scRNA-seq library preparation

Staged single cells were washed in three droplets of 50 μL L-15 supplemented with 1 mg/mL PVA within each droplet and transferred to a 0.2 mL skirted 96-well PCR plate (Thermo Fisher Scientific) containing 10 μL buffer RLT (Qiagen) with 1% 2-mercaptaethanol. Cells were lysed at room temperature for 5 min before centrifuging at 800 ***g*** for 30 s and were snap-frozen in liquid nitrogen for transcriptional profiling. In total, 30–32 oocytes of each stage and 52–57 somatic cells (pre-granulosa or granulosa cells) of each stage (primordial, transitioning, primary, and secondary follicle stages) were collected. Cells were compiled from independent collections. Single-cell RNA-sequencing (scRNA-seq) libraries were generated using SMART-Seq2 protocol (Trombetta *et al.* 2014). Briefly, cDNA was reversed transcribed from single cells using Maxima RT (Thermo Fisher Scientific) and whole transcriptome amplification (WTA) was performed. WTA products were purified using the Agencourt AMPure XP beads (Beckman Coulter, IN, USA) and used to prepare paired-end libraries with Nextera XT (Illumina, CA, USA). Single cells were pooled and sequenced on a NextSeq 550 sequencer (Illumina) using a 75 cycle High Output Kit (v2.5).

### scRNA-seq analysis and statistics

Raw sequencing data were demultiplexed and aligned to the GRCm38 genome using publicly available scripts on Terra (github.com/broadinstitute/TAG-public). Total gene counts and transcript per million (TPM) matrices were further analyzed in R (v.4.1.0) using the Seurat (v4) package (Hao *et al.* 2021). Cells expressing less than 500 genes were excluded from further analysis and genes were filtered to contain only those that were protein coding. To filter for high-quality cells for each category, we utilized the AddModuleScore function in Seurat v4 (Hao *et al.* 2021) for scoring marker gene expression modules established from published data sets (Pangas *et al.* 2006, Zhang *et al.* 2018, Tharp *et al.* 2020, Wang *et al.* 2020) (Supplementary Table 1). Oocytes that scored highly for somatic cell gene expression (>2 s.d. above the mean somatic cell module score) were removed. Similarly, somatic cells that scored highly for oocyte gene expression were also excluded (Supplementary Fig. 4). In order to account for the effects of integrating data from multiple sequencing runs, the scMerge package was used (Lin *et al.* 2019). Briefly, stably expressed genes were identified and leveraged as negative controls for normalizing batch effects present in the data. Initial clustering was performed on the filtered oocytes and somatic cells. Oocytes and somatic cells were then subset, re-normalized, re-scaled, and sub-clustered. Differentially expressed genes in the transitioning vs primordial stage samples were identified through DESeq2 (Love *et al.* 2014) with an adjusted *P* value cutoff of 0.05. Heatmaps are of top differentially expressed genes generated using the scaled data. Module scores for dissociation genes taken from Denisenko *et al.* were performed in Seurat. Gene ontology (GO) and pathway analysis were performed using two web-based portals, Metascape (Zhou *et al.* 2019) and Enrichr (Chen *et al.* 2013, Kuleshov *et al.* 2016, Xie *et al.* 2021) for GO biological process and GO molecular function, using the differentially expressed genes identified. Upstream regulator analysis was performed by Ingenuity Pathways Analysis (Qiagen IPA) on DESeq2 results generated. Target molecules were selected based on an adjusted *P* value less than 0.05, the activation z-score greater than 1.5 s.d., and endogenously expressed molecules.

## Results

### Animal-age- and oocyte size-dependent sample collection methods do not yield follicle stage-specific cell populations

To characterize follicle stage dynamics over the time course of neonatal murine ovary development, we quantified the proportion of follicle stages and measured oocyte diameter within ovaries collected in between P0 and P24, at animal ages that have typically been used in follicular cell isolation methods.

**Figure 2:**
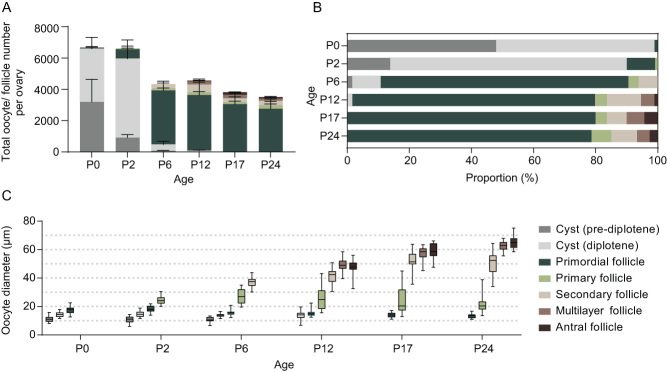
Oocyte and follicle-stage composition of neonatal murine ovaries at different ages. Oocytes before nest breakdown or from various follicle stages were quantified from post-natal day 0 (P0) to P24 CD-1 mice in stacked total number per ovary (A) and in proportion (B). Oocyte diameter from various stages was measured (C). Data represent mean with s.d. (A and B). Whiskers show min to max of the data points (C). *n*  =  5–6 ovaries (A and B) and 25–30 oocytes each group (C).

First, we quantified the total number of ovarian follicles in fixed ovarian sections from P0, P2, P6, P12, P17, and P24-aged CD-1 mice, as these time points are commonly used for collecting various follicular stage samples (Pan *et al.* 2005, Bristol-Gould *et al.* 2006) (Fig. 2A, B and Supplementary Fig. 5). At P0, females had over 6600 oocytes per ovary. More than 98% of the oocytes at this time point were still in ovarian cysts, and less than 2% had completed nest breakdown and were within individually encapsulated primordial follicles. No primary follicles were observed at P0. At P2, more oocytes were observed to have proceeded through the diplotene stage. The percentage of oocytes within primordial follicles increased to 10%, while 90% of oocytes remained in cysts. A few primary follicles were present at P2, indicating that the initial follicle activation occurs during P0-P2 in this mouse strain. By P6, the total follicle number dropped to 4300 per ovary, and primordial follicles became the predominant follicle class in the ovary, accounting for ~80% of the oocytes. Only 10% of oocytes remained in cysts at P6, and the remaining follicles were at the primary and secondary stages. The abundance of primordial follicles was observed to be stable between P6 and older ages, with relatively little change noted at P12, P17, and P24. Multilayer and antral follicles were observed at P12, and very few, if any, oocytes remained in cysts at this time. The proportion of each follicle class remained similar through P12 to P24.

We next measured the size of the oocytes within each follicle class in ovarian sections from mice ranging from P0 to P24. The diameter of oocytes in cysts was 11.2 ± 2.1–14.2 ± 1.8 μm (mean ± s.d.). The size of oocytes within cysts remained similar between P0 and P12. The diameter of primordial follicle oocytes ranged from 13.4 ± 1.4 to 18.3 ±2.1 μm, decreasing slightly from P0 through P24. Oocytes within primary follicles were 22.2 ± 6.4–27.2 ± 4.7 μm in diameter. Compared to oocytes within cysts and primordial follicles, oocytes within primary follicles had a much larger range of diameters at P6 and beyond. Oocytes within secondary follicles had a diameter of 36.8 ± 3.2–52.3 ± 6.2 μm from P6 to P24, and the size distribution was broader relative to oocytes of earlier stages. Oocytes within multilayer and antral follicles were 49.2 ± 4.5–62.4 ± 3.3 μm and 48.0 ± 5.0–64.9 ± 4.4 μm, respectively. The sizes measured showed a consistent increase in diameter from P12 to P24, as the size of whole follicles and the layer of somatic cells also increased (Fig. 2C).

Based on the range of oocyte diameter, there was an overlap in the size of oocytes within cysts and primordial follicles from P0 to P12. While smaller oocytes within primordial follicles overlapped with oocytes that remained in cysts, the larger oocytes within primordial follicles partially overlapped with those in primary follicles from P2 to P17. At P6, oocytes within primary follicles that were larger than 30 μm overlapped with those in secondary follicles. From P12 to P17, the size of oocytes within secondary follicles overlapped greatly with those in preantral and antral follicles (Fig. 2C). These data collectively demonstrate that the ovaries from mice at distinct ages commonly used to enrich for specific follicle stages possess heterogeneous follicle populations constituting multiple stages. Furthermore, there is a significant overlap in oocyte diameter between different follicle stages. Therefore, our results demonstrate that both animal-age- and oocyte size-specific follicle collection methods do not adequately result in the isolation of cell types from a single, pure follicular stage.

### Repeated enzymatic isolation protocol enables the reliable collection of precisely staged whole-follicle and single-cell samples

**Figure 3:**
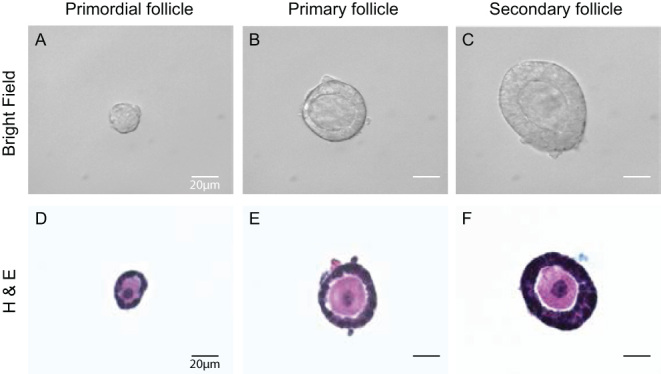
Follicle collection and imaging. Primordial, primary, and secondary follicles collected with the isolation method from P6 CD-1 mice (A, B, and C). The follicles were then fixed, encapsulated, and sectioned for H&E staining as shown in the lower panels (D, E, and F). Scale bar = 20 μm.

To collect stage-specific follicles and their associated single cells (oocytes and somatic cells), we developed a method that involves three rounds of enzymatic treatments followed by mechanical disruption of the ovaries and further enzyme treatment based on previous studies of our group and others (Fig. 1, see Materials and methods) (Kniazeva *et al.* 2015, Zhang *et al.* 2018). We applied our method to P6 ovaries to demonstrate reliable utility and specificity for primordial follicle collection. Ovaries were first incubated in liberase and DNase I before being mechanically disrupted with a trimmed pipette tip. Liberase digests extracellular matrix and aids the release of follicles from the ovary, while DNase I cleaves DNA released by dying cells and reduces cell clumping (Kniazeva *et al.* 2015). The procedure was repeated twice more with a shorter enzymatic incubation period and pipetted with trimmed pipette tips with smaller openings. It was crucial that the ovaries pass through the pipette tips easily during the pipetting step, as primordial follicles shed off the margin of the ovaries in layers (Supplementary Fig. 2). Therefore, the size of the tips should be adjusted accordingly to the size of the ovary. If starting off with a smaller tip size, the ovary tends to break apart into pieces without releasing individual primordial follicles. Upon collection, the follicular stage was validated by transmitted light microscopy and assessment of hallmark morphological criteria of somatic cells. For example, primordial follicles were identified as containing one single layer of squamous pre-granulosa cells, primary follicles as containing one layer of cuboidal granulosa cells, and secondary follicles as containing two layers of cuboidal granulosa cells (Fig. 3A, B, and C). We further confirmed the classification of isolated follicles by examining follicular architecture by histology (Fig. 3D, E, and F). Overall, we were able to routinely isolate around 800 primordial follicles (808.5 ± 128.8) per P6 ovary. The recovery rate was 23.3% when compared to the primordial follicle number quantified from fixed tissue sections. In addition to primordial follicles, around 17 primary follicles (17.2 ± 13.4) and 15 secondary follicles (15.6 ± 4.1) were collected from each P6 ovary (Table 1).

**Table 1:** Follicle number quantification with the isolation method. Total number of primordial, primary, and secondary follicles quantified from fixed sequential ovarian sections and follicles isolated freshly by the repeated liberase method across four to five trials. The recovery rate shows the amount of follicles in percentage one can collect from a P6 CD-1 ovary with the isolation method. Data represent mean ± s.d.

Follicle stage	Primordial follicle	Primary follicle	Secondary follicle
Follicle number (per ovary)	3464.5 ± 596.4	142.5 ± 22.0	267.0 ± 67.2
Freshly isolated follicle number (per ovary)	808.5 ± 128.8	17.2 ± 13.4	15.6 ± 4.1
Recovery rate	23.3%	12.1%	5.8%

**Figure 4:**
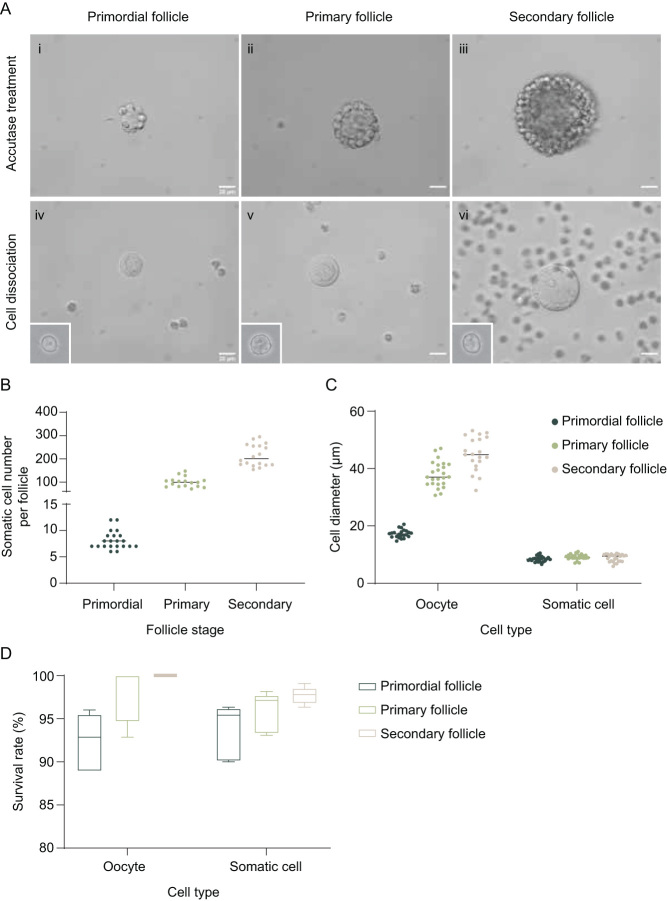
Single-cell collection and characterization. Primordial, primary, and secondary follicles were collected with the isolation method from P6 CD-1 mice and then incubated in Accutase solution for 5 (for primordial and primary follicles) to 10 min (for secondary follicle) for single-cell collection (A, i, ii, and iii). After the incubation, single oocytes and somatic cells (insets showing single somatic cells) were dissociated by mechanical disruption using pipettes of various sizes (A, iv, v, and vi). The number of the somatic cells of individual-staged follicles was quantified after single-cell isolation (B). The diameter (C) and the survival rate (D) of the oocytes and somatic cells were as well measured after cell isolation. Scale bar = 20 μm. *n*  =  18–21 follicles (B), 20–26 oocytes or somatic cells, and 4–5 independent experiments with 20 oocytes and 300 somatic cells (D).

To further collect single oocytes and somatic cells from staged follicles, follicles were incubated in accutase solution which cleaves junction proteins in between cells (bottom of Fig. 1) (Zhang *et al.* 2018). In accutase, somatic cells detached from neighboring somatic cells (Fig. 4A (i, ii, and iii)) after 5-10 min of incubation. The somatic cells do not fall off the oocytes spontaneously but require trituration with pipette tips of similar sizes to the follicles (Fig. 4A (iv, v, and vi)). Specifically, primordial follicles were pipetted with Pasteur pipettes of 20 μm, primary follicles were pipetted with 30 μm Pasteur pipettes, and secondary follicles were pipetted with 50 μm stripper tips. After cell dissociation, the number of individual somatic cells was quantified. A single primordial follicle contained 8.1 ± 1.7 pre-granulosa cells, a primary follicle contained 99.9 ± 21.4 granulosa cells, and a secondary follicle had 214.6±46.76 granulosa cells (Fig. 4B). The diameter of freshly isolated oocytes and somatic cells was then measured. The size of the oocytes aligned accordingly with numbers measured in fixed tissue sections, with primordial follicle oocytes averaging 17.3 ± 1.4 μm, primary follicle oocytes averaging 38.0 ± 4.4 μm, and secondary follicle oocytes averaging 45.1 ± 5.9 μm (Fig. 4C). Similar to what was observed in fixed ovarian sections, primary and secondary follicle oocytes had a larger size distribution than primordial follicle oocytes. On the other hand, pre-granulosa cells were no longer squamous after dissociating from the oocytes (Fig. 4A iv inset) and sized similarly to granulosa cells from primary and secondary follicles at 8.5 ± 0.9 μm, underlying the difficulty in separating the two morphologically if not staged beforehand during single-cell collection.

We next determined the cell survival rate of collected single cells after the repeated liberase and accutase method. With a dissection microscope, it is relatively easy to collect viable oocytes based on their intact and smooth membranes. However, it can be more challenging to determine if a somatic cell is viable or not. We incubated staged isolated cells with a live-dead dye and imaged the cells to quantify the survival rate. Our results showed that somatic cells of primordial, primary, and secondary follicles that were collected randomly for the assay demonstrated over 90% survival rate, and oocytes collected based on their intact membrane showed a 100% survival rate for secondary follicle oocytes, and over 90% survival rate for primordial and primary follicle oocytes (Fig. 4D). Overall, our method allows for the single-cell isolation of follicle stage-specific oocytes and somatic cells, with over 90% viability.

### Single-cell RNA-sequencing reveals pathways and upstream regulators involved in early follicle activation

scRNA-seq is one of the commonly utilized downstream methods for comprehensively characterizing the components of complex tissues and is critical for understanding the molecular signatures that drive follicle activation. Here, we performed scRNA-seq using established SMART-Seq2 protocols (Trombetta *et al.* 2014) on a total of 90 oocytes and 166 somatic cells collected using our liberase-accutase method which provided matched follicle-stage data (i.e. primordial, primary, and secondary) for each cell sequenced. This approach allowed us to tightly link follicle stage information with sequencing data, generate full-length transcriptomes, and is well suited for experiments aiming to profile hundreds rather than thousands of cells. Overall, we observed high-quality sequencing data across follicle stages. Specifically, the oocytes demonstrated a high number of unique genes detected per cell (>5K) with a low mapping rate to rRNA (<20%, Supplementary Fig. 6). Somatic cells showed lower detected genes (~3K) with a higher rRNA mapping rate (~30%), which is expected in smaller cells and agrees with prior sequencing studies on granulosa cells (Zhang *et al.* 2018). We additionally scored our data against dissociation-related genes that can be activated by cell dissociation (Denisenko *et al.* 2020) and show that regardless of the stage the scores are comparable and much lower in oocytes. This suggests that, while dissociation can certainly impact global transcriptomes, this is not a technical effect across follicle stages in our data (Supplementary Fig. 7). We next analyzed known oocytes and granulosa cells-specific genes and found that some were misexpressed (Supplementary Fig. 8). This leaky expression may have been due to the contamination of genes released from oocytes into the medium during sample collection. To exclude any potentially contaminated cells, we used a module scoring method based on target gene expression from published datasets (Supplementary Table 1) to remove somatic cells that expressed high oocyte markers, as well as oocytes that expressed high somatic markers, with a stringent cutoff of 2 s.d. (see Materials and methods). This allowed us to retain only the highest quality cells for further analysis.

**Figure 5:**
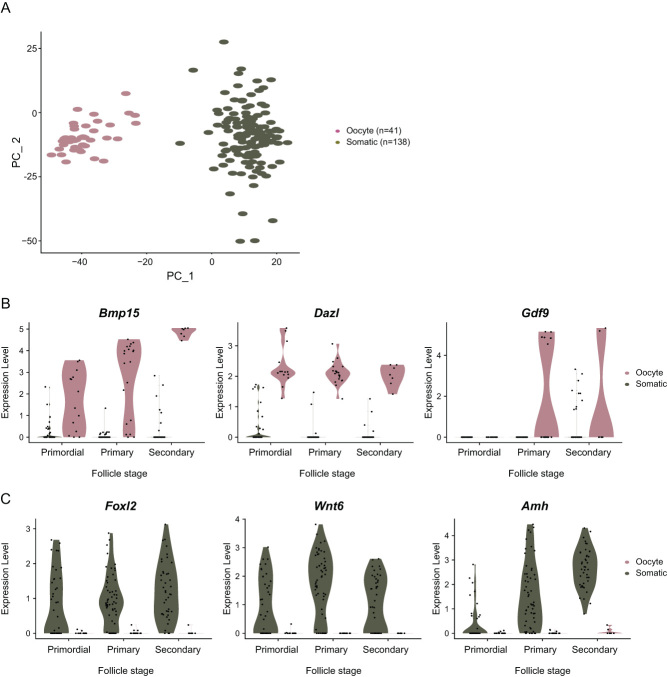
Gene expression dynamics and marker gene expression in the isolated single cells. Oocytes (14 primordial, 20 primary, and 7 secondary follicle stages) and somatic cells (40 primordial, 56 primary, and 42 secondary follicle stages) collected and filtered computationally were analyzed and visualized on a PCA plot after scRNA-seq. The cells were clustered and colored by cell type (A). Known oocyte (B) and somatic maker gene expression (C) of the three follicle stages were shown in the violin plots.

After sample filtering, 41 oocytes and 138 somatic cells were used for downstream analysis. A distinct separation between oocytes and somatic cells could be visualized upon principal components analyses (PCA) (Fig. 5A). Expression of known oocyte-specific genes, including *Bmp15 and Dazl*, was noted in primordial, primary, and secondary follicle stage oocytes, while *Gdf9* which is known to be expressed in growing oocytes, only increased after the primary stage (Fig. 5B). In somatic cells, genes including *Foxl2* and *Wnt6* were detected across multiple follicle stages, while *Amh* expression increased toward later stages (Fig. 5C).

**Figure 6:**
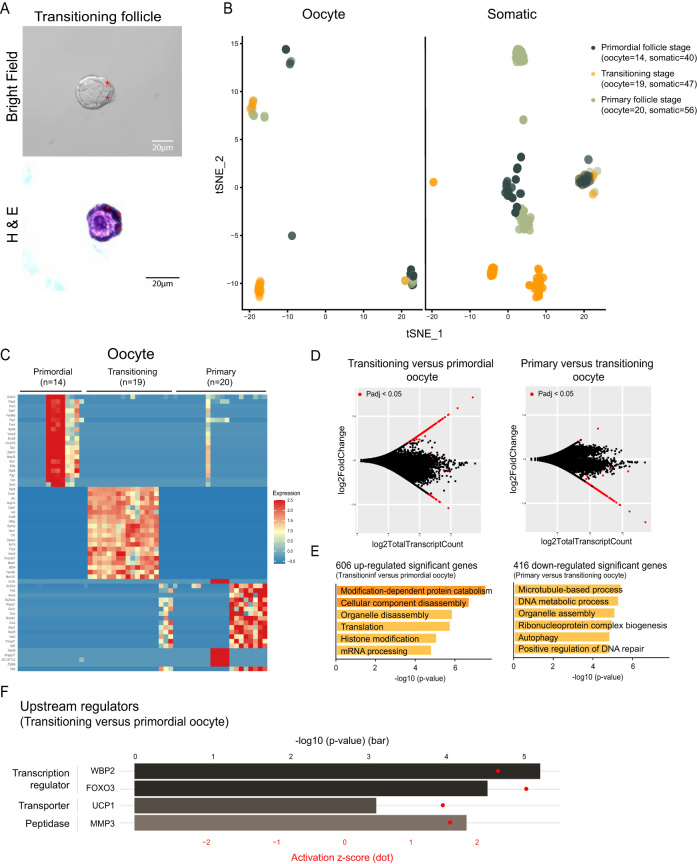
Gene expression dynamics in oocytes and somatic cells during follicle activation. Transitioning follicles were identified by partial squamous and partial differentiated somatic cells (wedge-shaped or cuboidal as marked in red asterisks) surrounding their central oocytes (A). The follicles were first imaged with a bright-field microscope (A, upper panel), then encapsulated and sectioned for H&E staining to verify the stages (A, lower panel). Single oocytes (14 primordial, 19 transitioning, and 20 primary follicle stages) and somatic cells (40 primordial, 47 transitioning, and 56 primary follicle stages) were analyzed and visualized on a t-SNE plot after scRNA-seq (B). The expression of top 20 genes with highest fold change value among primordial, transitioning, and primary follicle oocytes were selected and visualized on a heatmap (C). MA plots of transitioning vs primordial follicle oocytes (D, left) or primary vs transitioning oocytes (D, right) were shown with the log_2_ value of total transcript count on the x-axis, and the log_2_ value of fold change on the y-axis. Genes with significant adjusted *P*-value (*P*adj <0.05) were marked in red (D). Gene annotation analyses were performed using the upregulated DEGs identified in between transitioning oocytes vs primordial follicle oocytes (E, left), or downregulated DEGs identified in primary follicle oocytes vs transitioning oocytes (E, right). Upstream regulator analysis was performed on the DEGs identified in between transitioning oocytes vs primordial follicle oocytes. *P*-value was presented in bar plot according to the upper x-axis. Activation z-score was presented in dot plot according to the lower x-axis (F). Scale bar= 20 μm.

Most intriguingly, our isolation method allowed us to capture follicles that were phenotypically mid-transition between primordial and primary stage. These transitioning follicles were identified by having partial squamous and partial cuboidal somatic cells surrounding their central oocyte (Fig. 6A). We dissociated single oocytes and somatic cells from these transitioning follicles for scRNA-seq analysis. Interestingly, most transitioning oocytes separated from primordial follicle oocytes upon the t-distributed stochastic neighbor embedding (t-SNE) analysis, suggesting that transitioning follicle oocytes exhibit distinct transcriptional features from non-growing primordial follicle oocytes (Fig. 6B, left). This is also observed for transitioning somatic cells, which similarly clustered separately from somatic cells isolated from primordial follicles (Fig. 6B, right).

To better understand the differences in oocytes and somatic cells from primordial, transitioning, and primary follicles, we performed supervised differential expression analysis, selected the top 20 genes with the greatest fold change among each stage, and visualized the expression of these genes. First, in the oocytes, we observed a distinct gene expression pattern in the transitioning stage when compared to the primordial and primary stages (Fig. 6C). Target genes identified in the transitioning oocytes include genes reported to be involved in epigenetic, transcription, or translation regulation including *Dnmt1* (DNA methyltransferase 1), *Rlp10a* (Ribosomal protein L10a), *Eef1d* (Eukaryotic translation elongation factor 1 delta), *Tfcp2* (Transcription factor CP2), *Btf3l4* (Basic transcription factor 3 like 4), and *Fam46c* (Terminal nucleotidyltransferase 5C); genes related to nuclear transport and functions including *Nup214* (Nucleoporin 214), *Npm1* (Nucleophosmin 1), and *Tmem201* (Transmembrane protein 201); a ubiquitin gene *Ubc* (Ubiquitin C); a gene related to organelle movement in the oocyte *Padi6* (Peptidyl arginine deiminase 6); *Astl* (Astacin like metalloendopeptidase), which codes for an oocyte-specific oolemmal receptor (Fig. 6C).

When we compared transitioning and primordial stage oocytes, we identified 648 differentially expressed genes (DEG) (*P*adj <0.05), with the majority increased in expression in the transitioning oocytes (606 upregulated genes and 42 downregulated DEGs) (Fig. 6D, left) (Supplementary Table 2). GO analysis on the upregulated DEGs revealed GO biological processes and molecular function related to organelle disassembly, translation, and mRNA processing (Fig. 6E, left). Further, when comparing oocytes from transitioning vs primary follicles, the majority of DEGs (*P*adj <0.05) were downregulated in the primary follicle stage (416 downregulated and 8 upregulated genes) (Fig. 6D, right) (Supplementary Table 2). GO analysis revealed terms including microtubule-based process, organelle assembly, and autophagy to be enriched in genes downregulated in the primary follicle stage (Fig. 6D, right). Overall, this suggests that transitioning oocytes capture unique changes occurring *en route* to the primary follicle stage, with genes in transcription/ translation regulation and organelle reassembly increasing expression during the activation process.

**Figure 7:**
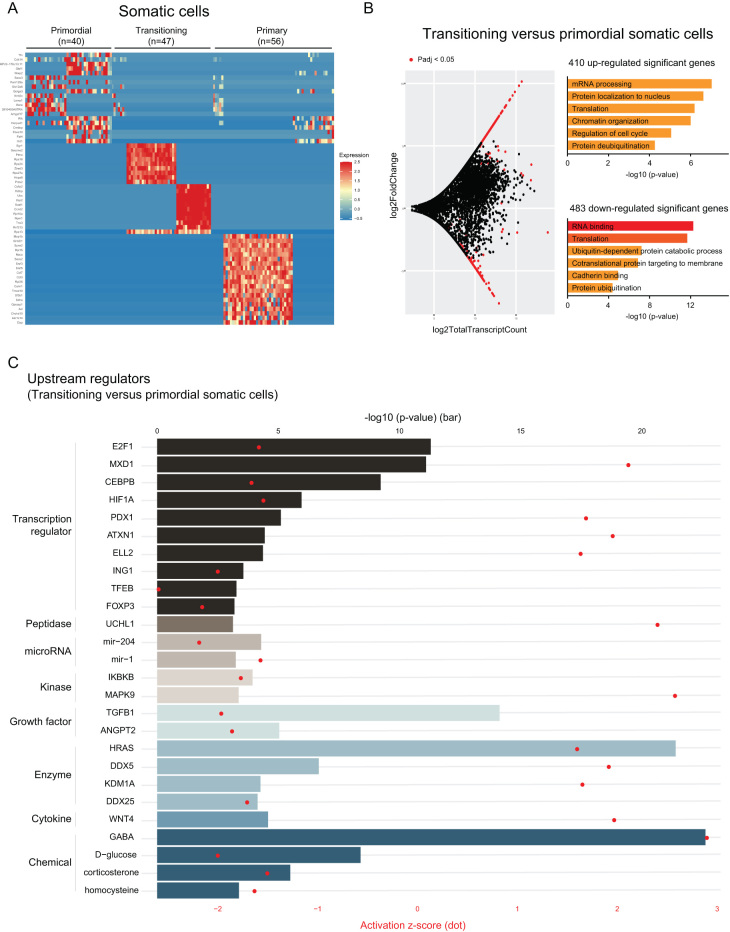
Gene expression dynamics and potential upstream regulators in transitioning somatic cells. The expression of top 20 genes with highest fold change value among primordial, transitioning, and primary follicle somatic cells were selected and visualized on a heatmap (A). MA plot of transitioning vs primordial follicle somatic cells (B, left) was shown with the log_2_ value of total transcript count on the x-axis, and the log_2_ value of fold change on the y-axis. Genes with significant adjusted *P*-value (*P*adj < 0.05) were marked in red (B, left). Gene annotation analysis (B, right) of transitioning vs primordial follicle somatic cells were performed using the identified upregulated DEGs (upper panel), or the downregulated DEGs in the transitioning stage (lower panel). Upstream regulator analysis was performed on the DEGs identified in between transitioning vs primordial follicle somatic cells. *P*-value was presented in bar plot according to the upper x-axis. Activation z-score was presented in dot plot according to the lower x-axis (C).

In the transitioning somatic cells, the expression profile of the top 20 genes with the greatest fold change revealed two major expression patterns with several ribosomal protein genes identified (*Rps16*, *Rps24*, *Rps27a*, *Rps13,* and*Rpl10a*) (Fig. 7A). This potentially reflects the different differentiation stages of the somatic cells (from squamous pre-granulosa cells to wedge-shaped and then to cuboidal granulosa cells). Meanwhile, primary follicle somatic cells, which contained all cuboidal granulosa cells, demonstrated the highest homogeneity in terms of gene expression pattern when compared to primordial and transitioning stages. The same degree of homogeneity is not seen in primordial stage somatic cells which also contained all squamous pre-granulosa cells (Fig. 7A). In contrast to the oocyte, the number of the DEGs that increased or decreased expression in the transitioning somatic cells when compared to the primordial stage were more comparable. In the total 893 DEGs identified, 410 increased and 483 decreased in expression in the transitioning somatic cells (Fig. 7B, left) (Supplementary Table 2). GO analysis revealed dynamic regulation in mRNA processing, translation, and protein localization in both the increased and decreased DEGs in transitioning somatic cells. Further, terms including cell cycle regulation and protein deubiquitination were enriched in upregulated DEGs, while cadherin binding and protein ubiquitination were enriched in the downregulated DEGs (Fig. 7B, right). During the progression from transitioning to primary stage follicle, there were 495 and 422 differentially up- and downregulated genes in the somatic cells, respectively. Many of these genes were enriched in RNA metabolism and chromatin organization (Supplementary Table 2).

Finally, to investigate potential upstream regulators of follicle transitioning, we performed upstream regulator analysis on transitioning oocytes and somatic cells, selecting only mammalian endogenous molecules with significant adjusted *P* values (*P*adj <0.05) and an activation z-score greater or less than ±1.5 for visualization. In the transitioning oocytes, we identified transcription factors WBP2 (WW domain-binding protein 2) and FOXO3 (Forkhead Box O3), as well as two that were not detected in our oocyte data set, transporter UCP1 (Uncoupling protein 1) and peptidase MMP3 (Matrix metalloproteinase 3), as potential upstream activators (Fig. 6F). On the other hand, a lot more targets were identified as potential upstream regulators in the transitioning somatic cells. Specifically, transcription regulators MXD1 (MAX dimerization protein 1), ATXN1 (Ataxin 1), peptidase UCHL1 (Ubiquitin C-terminal hydrolase L1), kinase MAPK9 (Mitogen-activated protein kinase 9), enzymes HRAS (H-Ras), DDX5 (Dead-box helicase 5), and KDM1A (Lysine Demethylase 1A), as well as cytokine WNT4 (Wnt family member 4) were among the potential upstream activators. Conversely, transcription factors including E2F1 (E2F transcription factor 1), HIF1A (Hypoxia-inducible factor 1 subunit alpha), TFEB (Transcription factor EB), miRNA mir-204 and mir-1, kinase IKBKB (Inhibitor of nuclear factor kappa B kinase subunit beta), growth factors TGFB1 (Transforming growth factor-beta 1) and ANGPT2 (Angiopoietin 2) were identified as potential negative upstream regulators of somatic transitioning (Fig. 7C). Our results corroborate prior studies that show significant roles of several of these targets in follicle activation, and we further suggest these targets as potential upstream regulators in the oocyte and the somatic cells upon follicle activation.

## Discussions

Historically, samples isolated using animal-age- or oocyte size-specific methods have been applied to the studies of ovarian follicle and oocyte development, for example, using different perinatal timepoints for collecting primordial or primary stage cells (Pan *et al.* 2005, Ford *et al.* 2021) or collecting size-specific oocytes to represent other follicular stages (Veselovska *et al.* 2015, Lv *et al.* 2021). These approaches allow for efficient sample collection and analysis in bulk quantity and have therefore established fundamental cellular events as well as identified critical players that drive follicle development. However, one limitation of age- or size-specific approaches is that they do not adequately represent a single pure follicle population as defined by the somatic cell compartment. For example, our results show that more than 98% of oocytes from CD-1 mice at birth remained in oocyte cysts. Nest breakdown, or the formation of individually encapsulated primordial follicles, does not peak until between P2 and P6 (Fig. 2). It should also be noted that there may be animal strain-specific differences. When the oocytes are dissociated from a perinatal ovary directly, the cytoplasmic bridges connecting the in-cysts oocytes disconnect, rendering it less likely to differentiate them from primordial follicle oocytes by transmitted light microscope, as oocytes from ovarian cysts and primordial follicles have similar morphological appearance and size. Our measurements also indicate that the size of primordial follicle oocytes can overlap with the size of primary follicle oocytes. These observations suggest that a precise characterization of a pure population can be more accurately achieved through first collecting individually staged samples, such as we achieved through transmission light microscopy imaging of all collected follicles (Hornick *et al.* 2012, Laronda *et al.* 2014, Kniazeva *et al.* 2015).

Follicle collection of primary and secondary follicle stages can be easily performed through mechanical disruption of the ovary (e.g. flicking insulin needles and pipetting (Shikanov *et al.* 2011)) which is effective in liberating follicles from the ovarian stroma with repeated agitation. However, the collection of individual primordial follicles requires gentle enzymatic dissociation, and their collection is more challenging than higher-staged follicles, due to their small size and their difficulty to manipulate and culture *in vitro*. Protocols for isolating primordial follicles have been described and compared by many research groups including in humans (Oktay *et al.* 1997, Abir *et al.* 1999, Dolmans *et al.* 2006, Vanacker *et al.* 2011, Lierman *et al.* 2015, Chiti *et al.* 2017, Zhang *et al.* 2018), primate (Hornick *et al.* 2012) and porcine ovaries (Greenwald and Moor 1989, Shi *et al.* 2007, Fattahi *et al.* 2020), yet it is much less described with murine samples. Based on a protocol that our group has previously published (Kniazeva *et al.* 2015), we report and quantify in this current study a detailed method for the collection of mouse primordial follicles, and their dissociation into single oocytes and pre-granulosa cells for the first time.

Follicles and single cells collected are suitable for various applications including live staining and imaging, encapsulation, fixation, and RNA extraction for transcriptomic analysis. With scRNA-seq, we demonstrated high sequencing quality of isolated oocytes and somatic cells using Smart-seq2, which enabled us to directly link cell information (e.g. type and stage) to full-length transcriptome data. Previous studies have performed sequencing on ovarian material with other methods, like 10X genomics. The main advantage of Smart-seq2 is that it is the most efficient method for analyzing fewer cells (Ziegenhain *et al.* 2017). For our specific application, given the low throughput and cells isolated at a given time, as well as the need to maintain a tight link between follicle stage and sequence data, Smart-seq2 (or other plate-based methods) was the preferred approach. Even though we did observe spillover of some cell-specific genes, likely due to the mRNA released into the medium from the dying cells during the isolation process, it is possible to apply more washing steps or to exclude these samples computationally by applying a cell type-specific module filter as performed here.

We were able to analyze transitioning stage oocytes and somatic cells and demonstrated distinct transcriptional programs within these cells. Our small sample size precludes the use of unsupervised methods to identify novel sub-clusters or sub-states of cells across stages, but our analyses that show that there is some overlap between stages suggest that this is worth pursuing in a much larger data set. At the onset of primordial follicle activation, partial pre-granulosa cells differentiate into mitotic cuboidal granulosa cells and become a transitioning follicle. During this change in somatic cell shape, junction proteins are extensively remodeled as the flattened pre-granulosa cells transform into cuboidal granulosa cells that are three to four-foldnarrower and six-fold taller (Da Silva-Buttkus *et al.* 2008, Mora *et al.* 2012). This process occurs morphologically prior to oocyte growth and FOXO3 nuclear exclusion in the oocyte (Hardy *et al.* 2018), which is a well-established marker of follicle activation (John *et al.* 2008). Once the follicle has fully transitioned into a primary follicle, organelles in the oocytes including the ER and Golgi rearrange and disperse out in the cytoplasm, instead of aggregating together as observed in a non-growing oocyte (Pepling *et al.* 2007). Our results demonstrated that at the time as partial surrounding somatic cells undergo differentiation, the oocyte from these transitioning follicles has already demonstrated a distinct molecular signature than a primordial follicle oocyte, with genes involved in organelle disassembly and translation regulation driving this transition. As a transitioning oocyte further progresses through the primary follicle stage, a major downregulation of genes enriched in organelle assembly and microtubule-based processes was subsequently observed. Moreover, other than dynamic changes in RNA and protein regulation, we also observed an enrichment of targets involved in cadherin binding, which likely reflected changes in cell shape during somatic differentiation. Interestingly, among the genes with the highest fold change of each stage, primordial and primary follicle oocytes demonstrated higher heterogeneity within the follicle stages, suggesting a potential ‘synchronized effect’ in the molecular signature of the transitioning oocytes during follicle activation (Fig. 6C).

The impacts of sample dissociation and sample handling have been well-documented for both bulk and single-cell studies (Denisenko *et al.* 2020), and in our work, we followed current best practices including minimizing the time from isolation to lysis, using the same method across biological replicates, and including batch variables for correction during analyses. Despite this, it should be noted that immediate-early responses may occur during isolation, and therefore, experiments should be performed with control groups and identified targets verified through secondary method (e.g. on flash-frozen ovarian tissue).

The identification of early response genes provides us with an ideal opportunity to characterize potential upstream regulators during follicle transitioning, which likely initiates in the somatic compartments (Zhang *et al.* 2014, Hardy *et al.* 2018). Upon upstream regulator analysis, we identified several potential upstream molecules that either positively or negatively regulate downstream target gene expression in the transitioning somatic cells. Several of these targets have been characterized in follicle activation. For example, studies have identified the importance of WNT signaling in pre-granulosa to granulosa cell differentiation (Ford *et al.* 2021, Habara *et al.* 2021). Further, TGFB1 signaling mediates cell cycle arrest in pre-granulosa cells (Hardy *et al.* 2018, Granados-Aparici *et al.* 2019), and inhibiting TGFB1 signaling promotes follicle activation (Wang *et al.* 2014). Moreover, oxygen level and blood vessel accessibility have been associated with follicle activation status (Shimamoto *et al.* 2019, Komatsu & Masubuchi 2020). Likewise, we observed HIF1A and Angiopoietin 2 as potential negative regulators in somatic differentiation (Baddela *et al.* 2020). Finally, female infertility has been implicated in transcription factor CEBPB deficient animals (Sterneck *et al.* 1997). In the transitioning oocyte, several DEGs that increased expression were identified as potential downstream targets of transcription factors FOXO3 and WBP2, a WW domain-containing protein that binds YAP (Yes-associated protein 1)/TAZ (Transcriptional coactivator with PDZ-binding motif) in the Hippo signaling pathway (Chen *et al.* 2019). Both FOXO3 (John *et al.* 2008) and the Hippo pathway (Kawamura *et al.* 2013, Kawashima & Kawamura 2018) are well-characterized players governing primordial follicle activation. Further analyses are required to determine the action of FOXO3 and WBP2 on downstream target genes during the initiation of oocyte transitioning, as FOXO3 is not yet fully translocated to the oocyte cytoplasm at the onset of somatic differentiation (Hardy *et al.* 2018).

Overall, we describe in this study a detailed isolation method for live primordial follicles and their dissociated single oocytes and pre-granulosa cells from neonatal murine ovaries. This has provided opportunities to study fundamental questions that directly compare cells at various stages along folliculogenesis and to investigate early cellular events during follicle transitioning that may be relevant to fertility preservation and reproductive longevity.

## Supplementary Material

Table 1. List of marker genes used to create module scores for oocytes and somatic cells.

Table 2. Gene ontology analysis.

Supplementary Figure 8. Oocyte and somatic modules for sample filtering. Oocyte and somatic modules were created using target genes listed in Supplementary Table 1 (see Method section). The upper and the lower panels show module score distribution of oocytes and somatic cells (GC) before and after sample filtering respectively.

Supplementary Figure 1. Representative images of oocytes and follicles of various stages. Oocytes and follicle number were quantified on ovarian sections of post-natal day 0, 2, 6, 12, 17, and 24 (P0 - P24) CD-1 animals. The red arrows mark pre-diplotene stage oocytes remained in cysts on the P0 section, while the yellow arrows mark diplotene stage oocytes remained in cysts. The rest of the yellow arrows mark primordial follicles on the P2 section, primary follicles on the P6 section, a secondary follicle on the P12 section, a multilayer follicle on the P17 section, and an antral follicle on the P24 section. Scale bar = 50 μm (P0 - P12) or 100 μm (P17 - P24). See Materials and methods for the criteria of classification.

Supplementary Figure 2. Illustration of the ovary during follicle isolation steps. Images representing a freshly dissected P6 CD-1 ovary before enzyme treatment (Fresh), after first round of enzyme treatment and trituration (First round), after second round of enzyme treatment and trituration (Second round), and the final round of enzyme treatment and trituration (Third round). Media from all three rounds of treatment were collected and passed through a cell strainer for follicle collection (Flow through). Red arrows mark the primordial follicles in the flow through media. Scale bar = 300 μm.

Supplementary Figure 3. Cell viability of collected single cells. Cell survival rate was quantified by commercial Live and Dead Dye on isolated oocytes and somatic cells, with green showing the living, red showing the dead cells. Scale bar = 200 μm.

Supplementary Figure 4.Cell filtering workflow. To obtain high-quality samples, cells were filtered initially with a cutoff of a minimum expression of 500 genes. Cells were then filtered based on the expression level of the marker genes from published datasets. Oocytes that scored highly for somatic cell gene expression (>2 standard deviations above the mean somatic cell module score) were removed. Similarly, somatic cells that scored highly for oocyte gene expression were also excluded. The four numbers in the parenthesis indicate the number of oocytes or somatic cells from primordial, transitioning, primary, and secondary stages.

Supplementary Figure 5. Oocyte and follicle-stage composition of neonatal murine ovaries at different ages. Oocytes before nest breakdown or from various follicle stages were quantified from post-natal day 0 (P0) to P24 CD-1 mice in total oocyte/ follicle number per ovary. N= 5-6 ovaries.

Supplementary Figure 6. scRNA-seq quality verification. The left panel shows total unique genes detected, and the right panel shows the mapping rate to rRNA in the individual oocytes and the somatic cells (GC) prior to sample pre-processing.

Supplementary Figure 7. Module scores for dissociation-related stress genes across follicle stages in our dataset for oocytes and somatic cells. Stages are in order from primordial, transitioning, primary, and secondary. Data is shown for oocytes (left) and somatic cells (right).

## Declaration of interest

A K S reports compensation for consulting and/or SAB membership from Merck, Honeycomb Biotechnologies, Cellarity, Repertoire Immune Medicines, Ochre Bio, Third Rock Ventures, Hovione, Relation Therapeutics, FL82, Empress Therapeutics, Dahlia Biosciences, intrECate Biotherapeutics, and Senda Biosciences. B A G reports compensation for consulting for FL82.

## Funding

This work was supported in whole or in part, by the Bill & Melinda Gates Foundation [INV-003385 and INV-010486]. Under the grant conditions of the Foundation, a Creative Commons Attribution 4.0 Generic License has already been assigned to the Author Accepted Manuscript version that might arise from this submission. Graphics in Fig. 1 was created with BioRender.com under BioRender’s academic license terms and the agreement number CA232AHZ2N.

## Author contribution statement

Y C, F E D, A K S, B A G, and T K W designed the experimental plan. Y C, D D R, R S D, B A G performed the experiments and analyzed the data. Y C, D D R, F E D, B A G, and T K W wrote the manuscript. All authors discussed the results and edited the manuscript.
