# Supplementary material for: Single-cell transcriptomics of staged oocytes and somatic cells reveal novel regulators of follicle activation
Source: Reproduction. 2022 Jun 17;164(2):55–70. doi: 10.1530/REP-22-0053 (PMC9354060; doi:10.1530/REP-22-0053)
Supplement: Table 1. List of marker genes used to create module scores for oocytes and somatic cells. [file supplementary_table_1.pdf]

Supplementary Table 1

Oocyte	Bmp15, Dazl, Ddx4, Dppa3, Figla, Gdf9, Lhx8, Nobox, Npm2, Oog1, Pou5f1, Sohlh1, Sox30, Sub1, Sycp3, Taf7l, Ybx2, Zar1, Zp2
Somatic cell	Aard, Aldh1a2, Amh, Amhr2, Cyp11a, Cyp19a1, Foxl2, Fshr, Fst, Gatm, Gng13, Hmgcs2, Inha, Inhba, Kitl, Krt8, Krt19, Rspo1, Star, Upk3b, Wnt6

Supplementary Table 1. List of marker genes used to create module scores for oocytes and somatic cells.
