# Supplementary figures and images for: Single-cell transcriptomics of staged oocytes and somatic cells reveal novel regulators of follicle activation
Source: Reproduction. 2022 Jun 17;164(2):55–70. doi: 10.1530/REP-22-0053 (PMC9354060; doi:10.1530/REP-22-0053)

P0

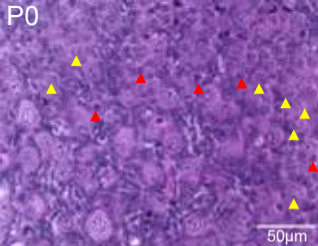

P2

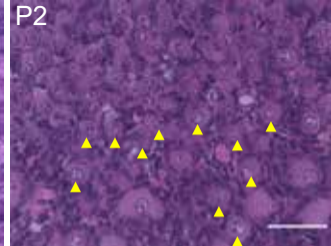

P6

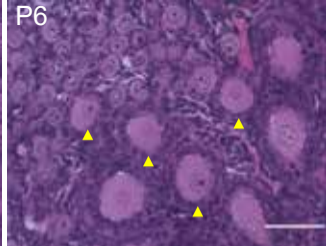

P12

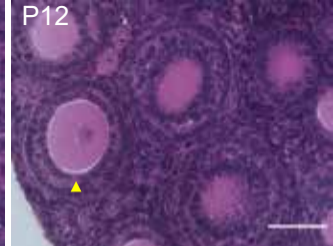

P17

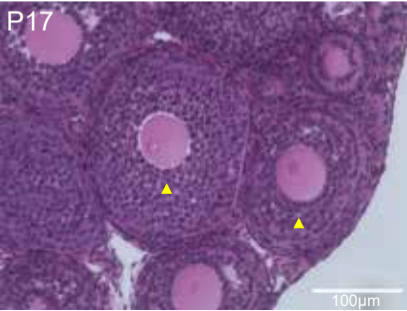

P24

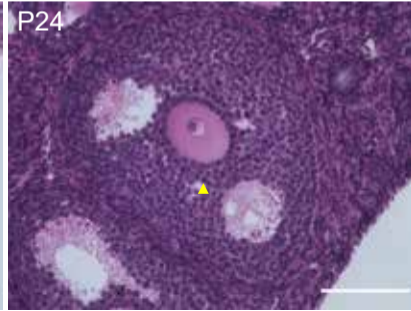

Supplement: Supplementary Figure 8. Oocyte and somatic modules for sample filtering. Oocyte and somatic modules were created using target genes listed in Supplementary Table 1 (see Method section). The upper and the lower panels show module score distribution of oocytes and somatic cells (GC) before and after s [file supplementary_figure_1.pdf]

Fresh

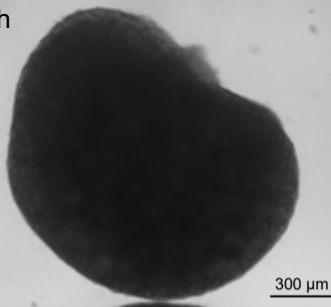

First round

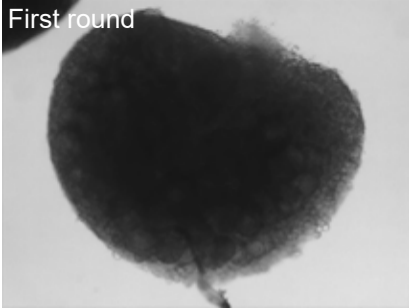

Second round

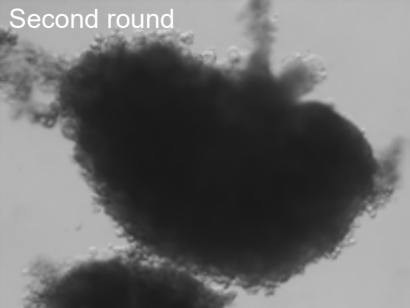

Third round

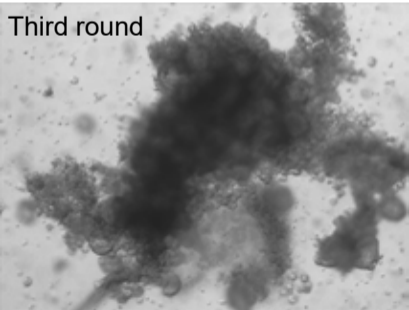

Flow through

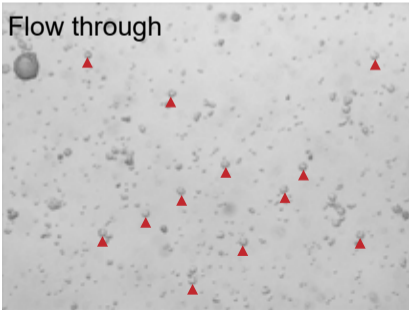

Supplement: Supplementary Figure 1. Representative images of oocytes and follicles of various stages. Oocytes and follicle number were quantified on ovarian sections of post-natal day 0, 2, 6, 12, 17, and 24 (P0 - P24) CD-1 animals. The red arrows mark pre-diplotene stage oocytes remained in cysts on the P0 sec [file supplementary_figure_2.pdf]

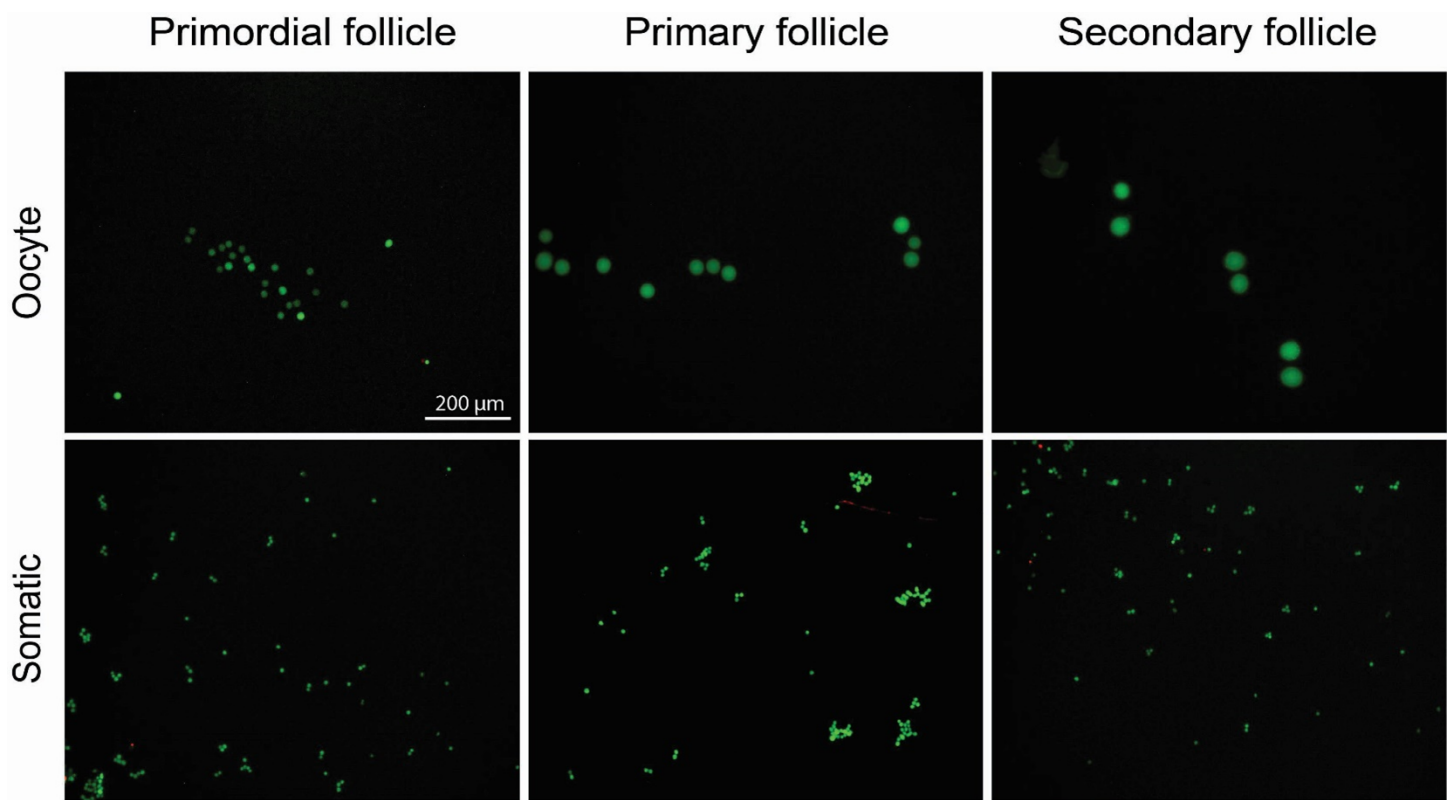

Supplement: Supplementary Figure 2. Illustration of the ovary during follicle isolation steps. Images representing a freshly dissected P6 CD-1 ovary before enzyme treatment (Fresh), after first round of enzyme treatment and trituration (First round), after second round of enzyme treatment and trituration (Secon [file supplementary_figure_3.pdf]

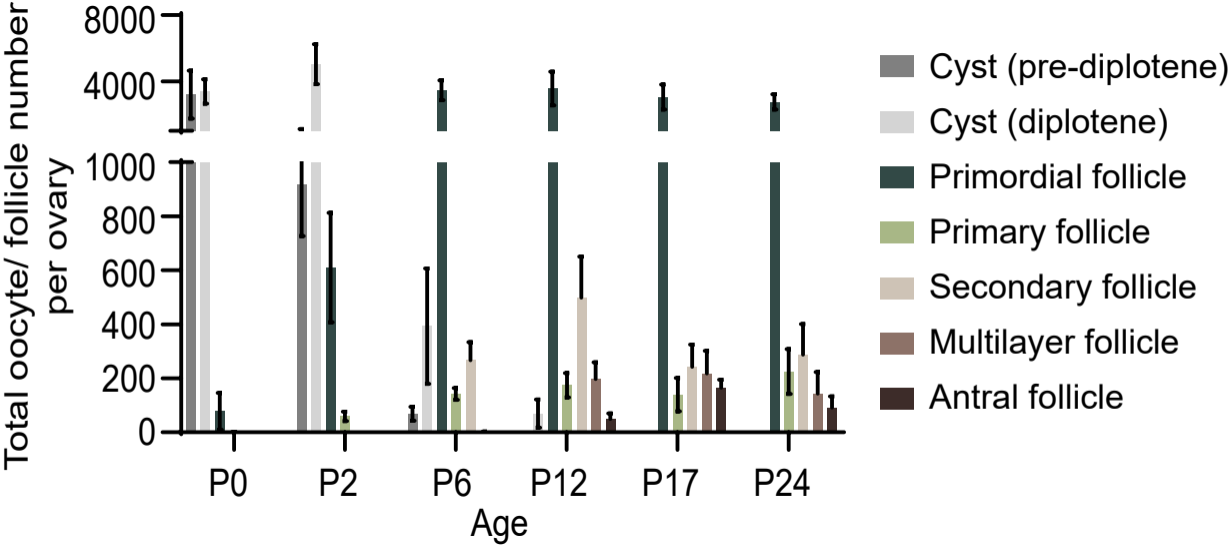

Supplement: Supplementary Figure 4.Cell filtering workflow. To obtain high-quality samples, cells were filtered initially with a cutoff of a minimum expression of 500 genes. Cells were then filtered based on the expression level of the marker genes from published datasets. Oocytes that scored highly for somatic [file supplementary_figure_5.pdf]

Total genes detected

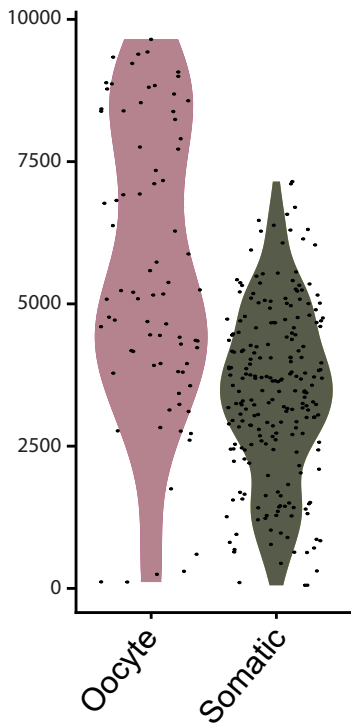

% rRNA

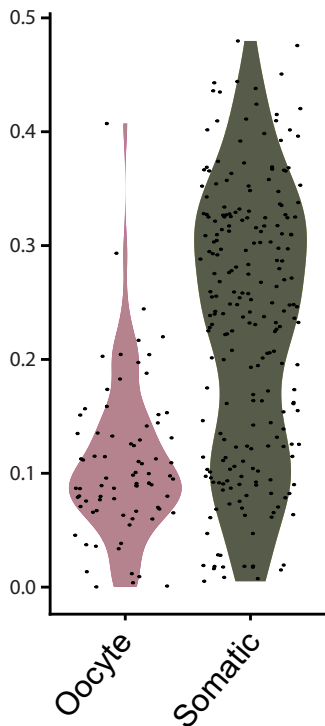

Supplement: Supplementary Figure 5. Oocyte and follicle-stage composition of neonatal murine ovaries at different ages. Oocytes before nest breakdown or from various follicle stages were quantified from post-natal day 0 (P0) to P24 CD-1 mice in total oocyte/ follicle number per ovary. N= 5-6 ovaries. [file supplementary_figure_6.pdf]

## Oocytes

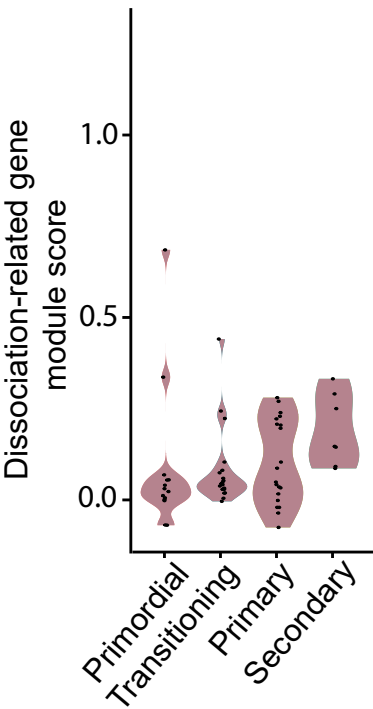

## Somatic cells

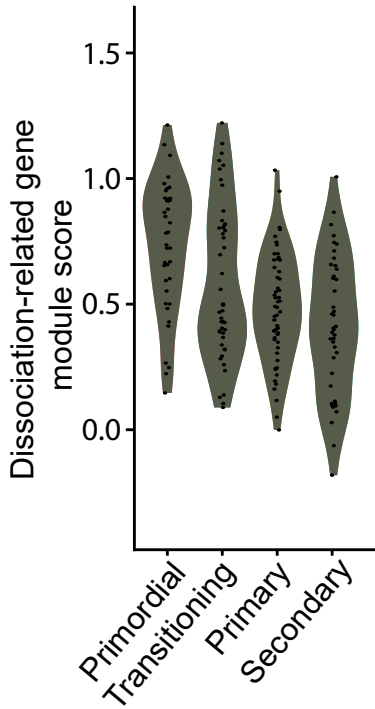

Supplement: Supplementary Figure 6. scRNA-seq quality verification. The left panel shows total unique genes detected, and the right panel shows the mapping rate to rRNA in the individual oocytes and the somatic cells (GC) prior to sample pre-processing. [file supplementary_figure_7.pdf]

Oocyte module

Before filtering

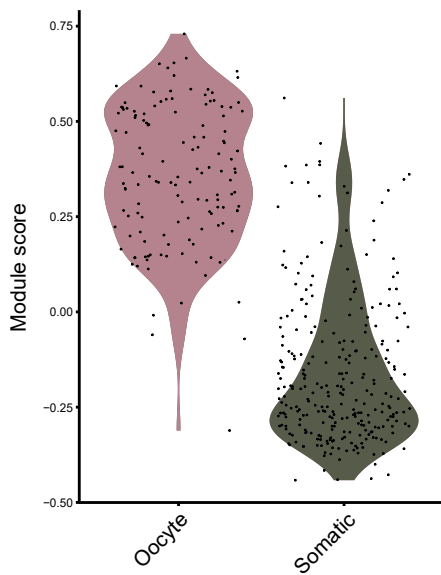

Somatic module

Module score

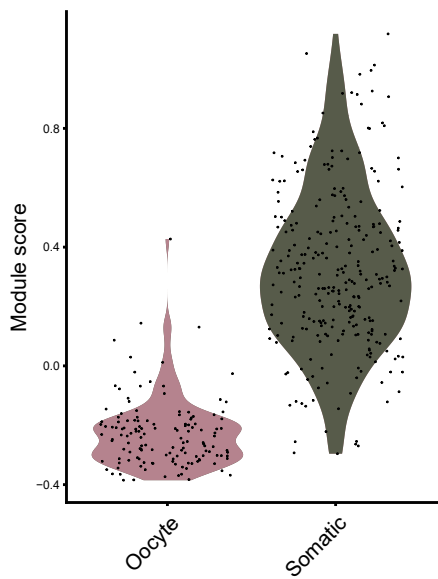

After filtering

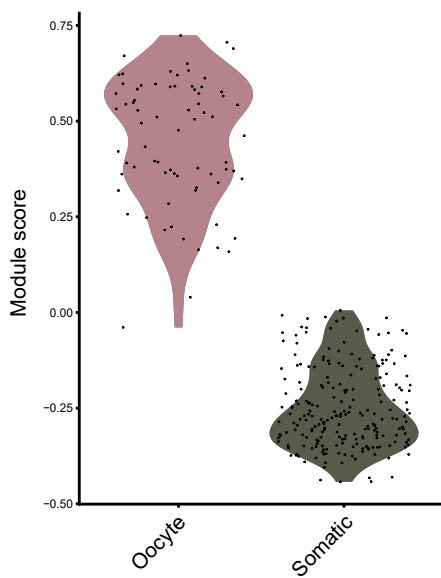

Module score

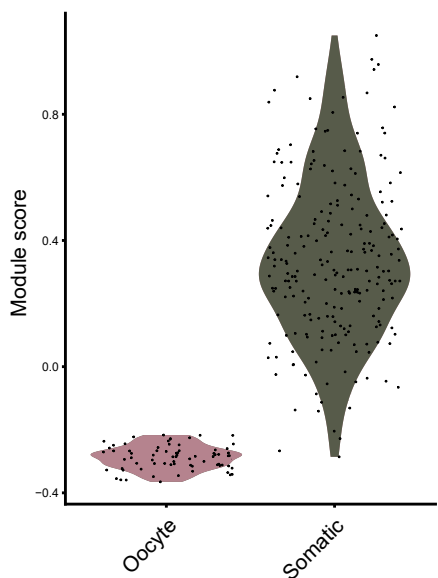

Supplement: Supplementary Figure 7. Module scores for dissociation-related stress genes across follicle stages in our dataset for oocytes and somatic cells. Stages are in order from primordial, transitioning, primary, and secondary. Data is shown for oocytes (left) and somatic cells (right). [file supplementary_figure_8.pdf]
