# Supplementary material for: Single-cell transcriptomics of staged oocytes and somatic cells reveal novel regulators of follicle activation
Source: Reproduction. 2022 Jun 17;164(2):55–70. doi: 10.1530/REP-22-0053 (PMC9354060; doi:10.1530/REP-22-0053)
Supplement: Supplementary Figure 3. Cell viability of collected single cells. Cell survival rate was quantified by commercial Live and Dead Dye on isolated oocytes and somatic cells, with green showing the living, red showing the dead cells. Scale bar = 200 μm. [file supplementary_figure_4.pdf]

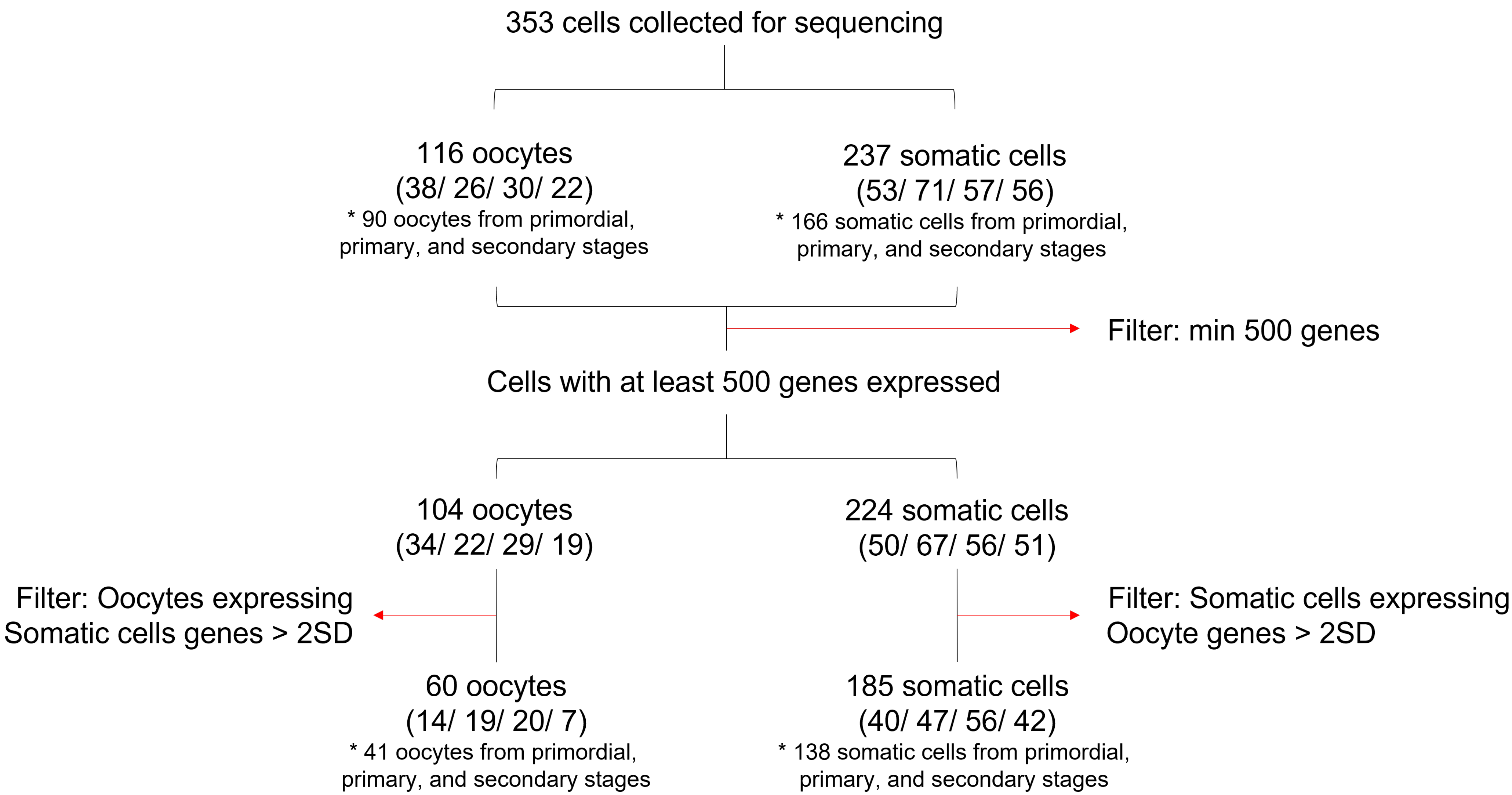
353 cells collected for sequencing
116 oocytes
(38/ 26/ 30/ 22)
* 90 oocytes from primordial, primary, and secondary stages
237 somatic cells
(53/ 71/ 57/ 56)
* 166 somatic cells from primordial, primary, and secondary stages
Filter: min 500 genes
Cells with at least 500 genes expressed
104 oocytes
(34/ 22/ 29/ 19)
224 somatic cells
(50/ 67/ 56/ 51)
Filter: Oocytes expressing Somatic cells genes > 2SD
Filter: Somatic cells expressing Oocyte genes > 2SD
60 oocytes
(14/ 19/ 20/ 7)
* 41 oocytes from primordial, primary, and secondary stages
185 somatic cells
(40/ 47/ 56/ 42)
* 138 somatic cells from primordial, primary, and secondary stages
